# Have Coping Resources and Trust in State Institutions Helped Jews and Arabs to Overcome Stress During the Iron Swords War?

**DOI:** 10.3390/ejihpe15040059

**Published:** 2025-04-13

**Authors:** Orna Braun-Lewensohn, Tehila Kalagy, Sarah Abu-Kaf

**Affiliations:** 1 Conflict Management & Resolution Program, Ben-Gurion University of the Negev, Beer-Sheva 8410501, Israel; ktehila@gmail.com; 2 Department of Public Policy & Administration, Ben-Gurion University of the Negev, Beer-Sheva 8410501, Israel

**Keywords:** coping resources, trust in state institutions, Jews and Arabs, psychological distress, war

## Abstract

The events of 7 October 2023 and the subsequent Iron Swords War have profoundly impacted Israeli society, leaving both Jewish and Arab populations exposed to unprecedented levels of violence and uncertainty. This study examined the roles of trust in state institutions and a sense of coherence (SOC) as coping resources that may mitigate psychological distress following exposure to such events. Four hundred and seventy-one participants (69.9% Jewish, 30.1% Arab) filled out a questionnaire that addressed the demographics, war exposure, trust in institutions, SOC, and mental-health outcomes. The Jewish participants reported greater exposure to war events, a stronger SOC, and less psychological distress than the Arab participants. Levels of trust in different institutions varied significantly between the groups, with Jews expressing greater trust in the military and Arabs reporting higher levels of trust in the parliament, educational system, and media. SOC protected against psychological distress among both groups. Trust in the military was associated with lower levels of distress among Jews, whereas trust in the parliament was linked to higher levels of distress among Arabs. This study highlights the need for tailored interventions that enhance the SOC and address disparities in institutional trust, to foster resilience among different sociocultural groups.

## 1. Introduction

### 1.1. Background and Aims

The events of 7 October 2023 took the state of Israel by complete surprise. Residents were left in shock at the scale of the hatred, brutality, and massacre that they experienced. A profound sense of abandonment emerged as entire communities felt forsaken—left without military protection and without state support. More than 1400 Israelis were murdered, 5600 were wounded (Peleg & Gendelman, 2023), and 250 individuals were kidnapped and taken into Gaza (Yadlin & Evental, 2024). This attack marked the beginning of the Iron Swords War. It was the first time since Israel’s establishment that the military failed to protect its civilians. Additionally, it was the first time that these communities were subjected to widespread evacuations. The events of 7 October represent an extreme and ongoing crisis unparalleled in Israeli history. The severity of the violence, coupled with the ongoing captivity of hostages in Gaza, has significantly eroded citizens’ sense of security and is expected to have long-term psychological consequences (Levi-Belz et al., 2024).

Given the unique circumstances of the 7 October attack and its aftermath, the current study aimed to fill critical gaps in the literature by examining the roles of trust in state institutions and the sense of coherence (SOC) in mitigating stress responses among Jewish and Arab populations in Israel. We specifically explored whether these factors contribute to mental-health outcomes in the face of extreme events and whether their effects differ across sociocultural groups.

### 1.2. Jews and Arabs in Israel

Diversity in Israel includes not only the variety of ethnicities that constitute the country’s overall population but also the cultural variety within the Jewish majority. Jewish society in Israel is composed of a variety of groups and identities that differ in their levels of affiliation with Judaism and religious involvement. Each of these streams has unique cultural, social, and religious characteristics, which influence daily life in Israel in different ways leading to ongoing discussions about identity, the public sphere, and the relationship between religion and state (Ben-Porat, 2016).

The Arab population in Israel comprises about 21% of the entire Israeli population. It includes Muslims (83%), Christians (9%), and Druze (8%; Central Bureau of Statistics, 2023). The society of this minority population differs from Jewish Israeli society in that it is less individualistic (Haj-Yahia, 2019). Arabs also differ from the Jewish majority in terms of language, religion, and other cultural factors (Al-Krenawi et al., 2009). They are a largely disadvantaged minority with major determinants of mental-health problems, including political, social, cultural, and economic factors (Abu-Kaf, 2019). The Arab minority is subject to various forms of discrimination that may contribute to social and economic disparities (Galilee Society, 2017; Haj-Yahya et al., 2022; Knesset Research and Information Center, 2016). This group suffers from poverty, harsh living conditions, violence, discrimination and stigma, and poor employment and working conditions (Central Bureau of Statistics, 2023). Thus, here is a growing lack of trust in the state, especially following the national conflict. The ongoing Israeli–Palestinian conflict has left Arab Israelis under significant pressure and with a sense of discrimination and mistrust (Popper-Giveon & Keshet, 2016).

### 1.3. Exposure to War

Exposure to war refers to the direct or indirect experience of violent conflict, which can include physical harm, the destruction of property, displacement, and the ongoing psychological toll of living in a conflict zone. In Israel, both Jewish and Arab populations have faced prolonged exposure to war, terrorism, and missile attacks, with significant mental-health implications. War exposure can be classified as objective exposure, which includes direct physical harm or witnessing violent events, and subjective exposure, which encompasses perceived threats and fears (Braun-Lewensohn & Sagy, 2011). One recent study compared the levels of war exposure among Jews and Arabs in the wake of the Iron Swords War. In that study, Jews reported higher levels of both direct and indirect (i.e., media) exposure, as compared to Arabs (Mayer et al., 2024). In a different study, researchers found indirect exposure to war to be related to acute stress disorder (Baziliansky & Sowan, 2025; Sowan & Baziliansky, 2024).

War exposure remains a significant determinant of mental-health outcomes among Jews and Arabs in Israel. In this paper, we explore the direct exposure of individuals from both of those groups to the war. We would like to add to the existing knowledge regarding the effects of war exposure on mental health among these two ethnic groups and examine war exposure alongside levels of trust in state institutions and the coping resource of the sense of coherence (SOC).

### 1.4. The Salutogenic Model and SOC

Antonovsky’s salutogenic theory (Antonovsky, 1987) focuses on the sources of health and emphasizes a central component known as SOC, which includes comprehensibility, manageability, and meaningfulness. Numerous studies, including studies of the diverse Israeli society, have indicated that SOC is significantly related to the ability to cope with stressful situations and maintain mental and physical health (e.g., Abu-Kaf et al., 2017). This concept has also been studied in the context of wars and among Jews and Arabs in Isreal. Research has shown that Jewish Israelis tend to report higher levels of SOC than their Arab counterparts (Abu-Kaf et al., 2017). Moreover, various studies have shown that, among both groups, the SOC serves as a protective factor in the context of mental-health outcomes (e.g., Abu-Kaf et al., 2017). Two studies have precisely measured the SOC as related to mental health among the various cultural groups in Israel during the Iron Swords War. Those studies concluded that the SOC is an important coping resource that has helped individuals to cope well with the stress of this ongoing war (Al-Said & Braun-Lewensohn, 2024).

### 1.5. Trust in State Institutions

Public trust in state institutions, the national security system, and national leadership is a key factor in national resilience, with a derivative impact on individual resilience. When levels of trust are high, social functioning and the ability of individuals to cope with crises improve (Kimhi, 2016). Therefore, trust in state institutions is an important resource for Israeli citizens as they cope with political threats (Herman et al., 2018). On the other hand, a lack of trust in state institutions or leadership can reduce resilience, leading to more symptoms of distress (Bodas et al., 2022; Marana et al., 2019).

In the Israeli context, especially among Jewish citizens, public trust in the Israeli Defense Forces (IDF) is usually high. In times of security threats, that trust tends to strengthen (Tiargan-Orr & Eran-Jona, 2016). During the Iron Swords War, several studies and reports have reported higher levels of trust in the IDF and the police than in the government (Herman & Anavi, 2023; Shapira & Elran, 2024). Due to their lower social status and the discriminatory policies toward the Arab population, low levels of trust in state institutions have been observed among that population. In a previous study that compared Jews and Arabs in terms of their trust in various state institutions, Jews consistently reported higher levels of trust (Mayer et al., 2024). Some studies have shown no gender differences in levels of trust in various state institutions (Xiao & McCright, 2015), whereas others have shown that males trust the state institutions more than females (Clayton et al., 2023). People of lower socioeconomic status have been shown to report lower levels of trust (Foster & Frieden, 2017).

As for the relationships between trust in different state institutions and various mental-health outcomes during this period, it has been persistently shown that higher levels of trust are negatively related to anxiety symptoms (Kaim & Bodas, 2024) and other negative mental-health outcomes, but only among Jewish individuals. These types of trust are not beneficial for Arabs, as these relationships are not prominent among that population group (Mayer et al., 2024).

The present study elaborates upon the existing knowledge regarding trust in various state institutions, namely the police, the parliament, the education system, the media, the court system, and the army. We sought to determine whether trust in different institutions affects mental health in the same way. Moreover, we wanted to examine the differences in trust and its relationships with mental health among the two large ethnic groups in Israel.

### 1.6. Emotional Reactions to Political Violence

Psychological distress is characterized by symptoms of anxiety and depression, which are common among individuals struggling to cope with significant threats or distress (Cénat et al., 2021). Studies examining the effects of human-made disasters, such as terror attacks and war, have documented a wide range of psychological distress (Lowell et al., 2018; Rigutto et al., 2021). As for the events of 7 October and the Irons Swords War, studies have found that the general Israeli population, across all sectors, has exhibited significant signs of distress (Marciano et al., 2024). A study that compared Jews and Arabs in this context found that Arab individuals tended to report higher levels of psychological distress (Mayer et al., 2024), which is consistent with the results of previous studies (e.g., Abu-Kaf et al., 2017; Achdut, 2024). Furthermore, women and younger individuals have also been found to report more stress (Kaim & Bodas, 2024).

In sum, this study aimed to explore how trust in state institutions and SOC serve as coping resources in mitigating stress responses among the Jewish and Arab populations of Israel. The uniqueness of this study lies in its examination of how these factors differ across socio-cultural groups (Jews and Arabs) in Israel, particularly levels of trust in various state institutions and their impact on psychological distress. By comparing these groups in terms of these critical variables, this study offers new insights into how war exposure, SOC, and trust influence mental-health outcomes in the wake of severe collective trauma. Our research questions and hypotheses were as follows:

1. Are there differences between Jews and Arabs in terms of the different variables, namely exposure to the events of 7 October, trust in state institutions, SOC, and psychological distress? We hypothesized that Jews would report more exposure to the events of 7 October, would express more trust in state institutions, and would report higher levels of SOC, whereas Arabs would report higher levels of psychological distress.
2. Are there relationships between the different variables among the two ethnic groups? Among both groups, we expected to find negative relationships between SOC and mental-health outcomes. We also hypothesized that there would be a negative relationship between trust in state institutions and mental-health outcomes among Jews, but no significant relationship between those factors among Arabs.
3. To what extent do the different sociodemographic variables (i.e., gender, age, socio-economic status), exposure to the events of 7 October and the subsequent war, trust in state institutions, and SOC, explain psychological distress among each of the sociocultural groups? We expected that women, younger participants, and individuals of a lower socioeconomic status would exhibit poorer mental health, whereas SOC would contribute to better mental health among both ethnic groups. Additionally, we expected trust in state institutions to be a protective factor that plays a significant role in the explanation of psychological distress only among the Jewish population.

## 2. Materials and Methods

### 2.1. Sample Description

A total of 471 participants completed questionnaires distributed over a period of 3 days, approximately 90 days after the 7 October attack. The questionnaires were disseminated via the Midgam panel (https://www.midgampanel.com/).

Participants ranged in age from 18 to 89 years, with a mean age of 40.82 (*SD* = 15.56). Jews comprised 69.9% of the sample; women comprised 50.7% of the sample. Socioeconomic status was examined in terms of income: 53.9% reported below-average income, 26.3% reported an average income, and 19.8% reported above-average income. χ^2^ testing was conducted to examine differences in demographic characteristics between the two groups. In terms of gender, no differences were found between the two groups: χ^2^ = 1.18, *p* > 0.05. Regarding socioeconomic status, χ^2^ = 35.61, *p* < 0.001, suggesting that the Jewish group had a higher socioeconomic status than the Arab group. The Arab population was a bit younger (*M* = 38.34 years, *SD* = 10.94) than the Jewish population (*M* = 41.90, *SD* = 0.94; *t* = 2.70, *p* < 0.05.)

### 2.2. Procedure

This study was approved by the Ethics Committee of the Conflict Management and Resolution Program at Ben-Gurion University of the Negev (Approved Ethics Form No. 2023-017). Participants were provided with information regarding the study’s objectives. They were also informed that their participation was voluntary and anonymous and that they were free to withdraw their participation at any time and for any reason.

### 2.3. Research Tools

The sociodemographic questionnaire included questions regarding participants’ gender, age, and socioeconomic status.

#### 2.3.1. War Exposure

Exposure to the events of 7 October and to the Iron Swords War was measured using 7 yes (2)/no (1) questions. Examples of items were as follows: were you evacuated from your home? Were any of your family members murdered/killed? Were any of your family members kidnapped? Did missiles fall in your town? The answers were summed to an index ranging from 7 to 14. Higher scores indicate greater exposure.

#### 2.3.2. Trust in State Institutions

Trust in state institutions was measured using six separate questions, one for each institution. The questions related to the police, the parliament, the educational system, the media, the court system, and the army. Each question was rated on a scale of 1 (*do not trust at all*) to 6 (*trust very much*). In order to understand the reported perceptions regarding each of the institutions, we evaluated the responses to each item separately.

#### 2.3.3. Sense of Coherence (SOC)

The SOC (Antonovsky, 1987) was measured using a series of semantic differential items scored on a 7-point Likert scale, with anchor statements at each end. Higher scores indicated a stronger SOC. In this study, SOC was assessed using a short scale consisting of 13 items. We calculated the mean of the responses to the13 items to create the scale. The reliability of the scale was examined: α = 0.81.

#### 2.3.4. Psychological Distress

The Brief Symptom Inventory (BSI; Derogatis & Fitzpatrick, 2004) was used to assess three domains of psychological and psychiatric distress: somatization, depression, and anxiety. This questionnaire consists of 18 items, rated on a 5-point Likert scale (0 = *not at all*; 4 = *very much*). We calculated the mean of the responses to the 18 items to create the mental-health/psychological-distress scale. The reliability for the overall scale was α = 0.93.

### 2.4. Data Analysis

First, before we analyzed the research questions, Chi-square tests and *t*-tests were conducted to examine differences in sociodemographic variables between the two research groups (Jews and Arabs). Then, we began to analyze the research questions. To address the first research question, a *t*-test was conducted. To address the second research question, Pearson correlation tests were performed. To address the third research question, a hierarchical regression was conducted for the two groups separately. In the first step of the regression, sociodemographic variables were entered. In subsequent steps, SOC and the various variables representing the levels of trust in different state institutions were entered into the model, to examine how they did or did not help to explain mental-health outcomes (i.e., anxiety, depression, somatization).

## 3. Results

The first research question concerned the differences between Jews and Arabs in terms of exposure to the events of 7 October and the Iron Swords War, as well as trust in different state institutions, the coping resource of the SOC, and the mental-health outcomes of anxiety, depression, and somatization. Significant differences were observed between the two groups for most of these variables. Jews had greater exposure to war events and reported a stronger SOC and fewer symptoms of psychological distress. Arabs reported greater trust in the parliament, the educational system, and media, whereas Jews had more trust in the army. Differences between the two groups are presented in Table 1.

The second question was related to the relationships among the study variables. Our results show that, between both groups, SOC was negatively related to psychological distress. However, other variables were related to mental health in different ways. For example, while war exposure was positively related to negative mental-health outcomes among the Jewish group, it was not associated with those mental-health outcomes among the Arab group. Differences also emerged in terms of levels of trust in different institutions. Among the Jewish group, only trust in the army was negatively related to the examined mental-health outcomes. Among the Arab group, on the other hand, trust in the educational system, the court system, and the army were all positively related to the examined mental-health outcomes. These results are presented in Table 2.

Following the discovery of differences between the two groups in the correlation analysis, two regression analyses were conducted—one for the Jewish group and one for the Arab group. The results of these analyses are presented in Table 3. Our results show that the entire model explained 40% of the variance in psychological distress among the Jewish group and 33% of the variance in psychological distress among the Arab group. The SOC, a personal coping resource, was the strongest predictor of psychological distress among both groups, explaining 22% of the variance in psychological distress among the Arab participants and 17% of the variance in psychological distress among the Jewish participants. Some major differences in the explanatory power of each step emerged. For example, sociodemographic variables explained 16% of the variance in psychological distress among the Jewish group but only 1% of the variance in psychological distress among the Arab group. It seems that, for the Jewish participants, gender and age played major roles. Moreover, among the Jewish group, war exposure played a significant role in explaining psychological distress, explaining 3% of the variance in that factor. Trust in the army and the media were also significant, adding an additional 4% to the explanation of variance. For the Arab group, only trust in the parliament played a significant role in explaining the variance in psychological distress. However, the trust items together added a total of 9% to the explained variance.

## 4. Discussion

In this study, we sought to examine the roles of trust in state institutions and SOC in relieving stress responses among Jewish and Arab populations in Israel following the events of 7 October 2023 and the Iron Swords War. Our findings highlight significant differences between the two groups in terms of exposure to war-related events, trust in various state institutions, SOC, and psychological distress. Additionally, the relationships between these different factors and mental-health outcomes differed between Jews and Arabs, shedding light on the unique sociopolitical and psychological dynamics affecting each group.

### 4.1. Ethnic Differences in Exposure, Trust, and Psychological Distress

Consistent with our hypothesis, Jewish participants reported higher levels of direct and indirect exposure to war-related events than their Arab counterparts. This aligns with previous research indicating that Jewish communities are often at the forefront of the military conflict in Israel, leading to increased exposure to war events (Mayer et al., 2024). Despite this heightened exposure, Jewish participants exhibited a stronger SOC and lower levels of psychological distress than Arab participants. This finding is in line with past research demonstrating that SOC serves as a key protective factor against negative stress-related psychological outcomes (Abu-Kaf et al., 2017).

Trust in state institutions also varied significantly between the two groups. Jews expressed greater trust in the military, whereas Arabs reported higher levels of trust in the parliament, educational system, and media. These differences likely reflect broader historical and political experiences. Jewish citizens traditionally perceive the military as a protective force. Part of the identification with the military and trust in it is because ethnic affiliation and the military will always reflect the political–military conflict. Most Jews are in some way part of this system and, therefore, they place more trust in it. The IDF is also called the “people’s army” and is part of the Jewish-Israeli ethos (Shafran-Gittelman, 2020), and its very existence among the majority group constitutes a threat to the minority. On the other hand, individuals from Arab society, as a distinct national and cultural minority that does not serve in the military, have less trust in that institution. Arab individuals may view political and educational institutions as more central to their identity and societal advancement (Herman et al., 2018). Notably, trust in the court system and the police did not significantly differ between the two groups, suggesting shared skepticism or confidence in these institutions.

The finding that Arabs, a historically marginalized and socioeconomically disadvantaged minority in Israel, reported higher levels of trust in the parliament, educational system, and media is particularly surprising. One possible explanation is that these institutions are perceived by Arabs as avenues for representation, advocacy, and potential societal change. The parliament, despite its limitations, includes Arab political parties that provide a platform for Arab concerns, which may contribute to a sense of trust. Similarly, the educational system may be seen as a means of upward mobility and integration into Israeli society, despite existing inequalities. The media, particularly Arabic-language outlets, may serve as a crucial source of information and community discourse, reinforcing trust in its role. However, this trust does not necessarily indicate full confidence in the institution’s fairness or effectiveness, but may reflect a pragmatic reliance on them as key societal mechanisms. It could be that wherever there is representation of members of the minority group, members of that group feel a sense of belonging and also that they have influence within that institution, and therefore, they place more trust in those institutions.

### 4.2. The Role of SOC in Mental Health

As hypothesized, SOC emerged as a robust protective factor against psychological distress among both Jews and Arabs, reinforcing Antonovsky’s (1987) salutogenic model. SOC was negatively correlated with distress among both groups, indicating that individuals who experience higher comprehensibility, manageability, and meaningfulness were better equipped to navigate the psychological challenges posed by war exposure. This finding is consistent with previous studies demonstrating SOC’s buffering effects on mental health during crises (Al-Said & Braun-Lewensohn, 2024; Braun-Lewensohn & Sagy, 2011).

### 4.3. Trust in State Institutions and Psychological Distress

Our results provide nuanced insights into the relationship between trust in state institutions and mental health. Among the Jewish group, trust in the army was negatively associated with psychological distress, reinforcing prior findings that public confidence in the military enhances resilience during national crises (Tiargan-Orr & Eran-Jona, 2016). In contrast, trust in other institutions, such as the police, parliament, and courts, was not significantly linked to any of the examined mental-health outcomes, suggesting that for Jewish individuals, institutional trust is primarily anchored in the military’s role in national security.

Among the Arab participants, trust in the educational system, courts, and army was positively correlated with psychological distress. This counterintuitive finding suggests that reliance on state institutions may not provide the same psychological benefits for Arabs as it does for Jews. One possible explanation is that greater trust in these institutions among Arabs may stem from a desire for systemic stability rather than actual confidence in institutional support during crises. Additionally, the longstanding history of discrimination and socio-political marginalization among Arab citizens (Knesset Research and Information Center, 2016) may lead to disillusionment, with trust in state institutions failing to translate into tangible coping benefits.

### 4.4. The Explanation of Psychological Distress by the Different Variables

Regression analyses further clarified the differential predictors of psychological distress among each ethnic group. Among both groups, SOC was the most important factor contributing to levels of psychological distress. Once again, SOC was found to be a significant resilience factor, which aids individuals from various ethnic groups and backgrounds to cope with stressful events. Apart from the SOC, several differences emerged. Among the Jews, sociodemographic factors (gender and age) explained a significant portion of variance in distress, with younger individuals and women reporting higher levels of distress, consistent with previous findings on vulnerability to stress in crisis situations (Kaim & Bodas, 2024). War exposure also played a significant role in explaining distress, reaffirming the traumatic impact of direct exposure to violence (Marciano et al., 2024). Additionally, trust in the army and the media significantly contributed to distress levels, with greater trust in the army serving as a protective factor while trust in the media served as a stressor.

Among the Arabs, sociodemographic factors played a minimal role in predicting psychological distress, while trust in the parliament was the only institutional trust variable significantly associated with mental-health outcomes. This suggests that, for Arabs, in the context of war, psychological distress is less influenced by demographic characteristics.

### 4.5. Limitations

Despite its contributions, this study has several limitations. First, the cross-sectional design of this study limits our ability to draw causal inferences among trust, SOC, and psychological distress. Future research should employ longitudinal designs to assess changes over time. Second, self-reported measures may introduce biases, such as social desirability or recall bias. Third, the study sample, though diverse, may not fully represent all subgroups within the Jewish and Arab populations. Lastly, the summer of 2023 was marked by unprecedented demonstrations against the Israeli government over the constitutional amendments. This could have influenced people’s reaction to the acute stressful situation of 7 October and the Iron Swords War. Since we did not check the participants based on our variables prior to 7 October we cannot evaluate if and in what ways it had an effect.

### 4.6. Implications and Future Directions

The findings of this study have important theoretical and practical implications. On the theoretical level, our results contribute to the growing body of literature on coping mechanisms in conflict-ridden societies, particularly by elucidating ethnic-based differences in the roles of SOC and institutional trust. Practically, these insights can inform policymakers and mental-health professionals as they tailor interventions that enhance psychological resilience. Efforts to strengthen SOC—through community-based programs, educational initiatives, and mental-health support—may prove beneficial for both Jews and Arabs.

Furthermore, our findings highlight the need to address disparities in institutional trust and its implications for mental health. For Jewish individuals, strengthening trust in the military may continue to serve as a protective factor, whereas for Arabs, addressing systemic inequalities and enhancing the perceived legitimacy of state institutions could foster greater psychological resilience. Future research should explore additional socio-demographic variables (such as political affiliation, religiosity/secularism, and possibly geography), as well as other variables, such as social support, political ideology, and personal experiences with discrimination that may mediate or moderate the relationship between institutional trust and mental health.

In conclusion, this study underscores the complex interplay among exposure to political violence, coping resources, and mental health among different ethnic groups. While SOC remains a universal protective factor, the role of institutional trust in psychological distress differs significantly between Jews and Arabs. Addressing these disparities through targeted interventions may contribute to greater national resilience and social cohesion in Israel’s multiethnic society. The paper contributes to the field by enhancing our understanding of intragroup differences within Israel regarding perceptions of key institutions, such as the military and parliament. Furthermore, an intriguing finding of this study was the variation in trust levels across different societal groups, particularly the relatively lower trust in governmental institutions among the Jewish population. This contrasts with existing literature that often emphasizes Arab citizens’ distrust in the state. One possible explanation lies in the dynamic political climate and shifting public discourse in Israel, which may have influenced perceptions of institutional reliability among Jewish subgroups. Additionally, the role of socio-economic status and ideological affiliation may be more significant than previously assumed, with individuals from marginalized or politically disillusioned groups exhibiting lower trust regardless of ethnic identity. Another unexpected result was the relatively high level of trust in the military across most groups, despite political and social tensions. This could be attributed to the military’s unique role in Israeli society as a unifying institution that transcends political divisions, reinforcing a collective sense of security and national resilience. These findings suggest that trust in institutions is not solely dictated by ethnic or religious identity but is shaped by a complex interplay among socio-political experiences, historical narratives, and shifting public sentiments. Future research should further explore these dynamics to refine our understanding of how institutional trust is constructed and challenged in diverse societal contexts.

## Figures and Tables

**Table 1:** Differences between Jews and Arabs in terms of the main study variables.

	Jews *N* = 328 *M* (*SD*)	Arabs *N* = 143 *M* (*SD*)	*t*
Exposure to war events (7–14)	9.48 (1.33)	8.65 (1.14)	6.88 ***
SOC (1–7)	4.64 (0.91)	4.08 (0.80)	6.23 ***
Trust in the police (1–6)	3.71 (1.37)	3.94 (1.73)	−1.51
Trust in the parliament (1–6)	2.39 (1.34)	4.61 (1.49)	−15.72 ***
Trust in the educational system (1–6)	3.12 (1.31)	3.66 (1.54)	−3.55 ***
Trust in the media (1–6)	2.97 (1.53)	4.16 (1.43)	−7.75 ***
Trust in the court system (1–6)	3.34 (1.62)	3.35 (1.62)	−0.05
Trust in the army (1–6)	4.84 (1.18)	3.22 (1.92)	9.25 ***
Psychological distress (0–4)	1.08 (0.72)	1.32 (0.87)	−3.08 ***

*** *p* < 0.001.

**Table 2:** Correlations between the different study variables among the two population groups (blue—Jews; red—Arabs).

	Exposure	SOC	TP	TPA	TE	TM	TC	TA	PD
Exposure	1	0.05	−0.20 **	−0.03	−0.17 *	0.10	0.19 *	0.20 **	−0.06
SOC	−0.10	1	0.22 **	0.10	−0.12	−0.22 **	−0.16	−0.33 ***	−0.51 ***
TP	0.01	0.12 *	1	0.62 ***	0.41 ***	0.52 ***	0.36 ***	0.72 ***	0.13
TPA	−0.02	0.11	0.46 ***	1	0.49 ***	0.54 ***	0.40 ***	0.48 ***	−0.04
TE	0.04	0.16 **	0.48 ***	0.57 ***	1	0.54 ***	0.62 ***	0.47 ***	0.17 *
TM	−0.16 **	0.10	0.33 ***	0.14 *	0.34 ***	1	0.62 ***	0.63 ***	0.10
TC	−0.12 *	0.17 **	0.30 ***	0.05	0.29 ***	0.66 ***	1	0.53 ***	0.19 *
TA	−0.04	0.21 ***	0.46 ***	0.15 **	0.31 ***	0.17 **	0.21 ***	1	0.20 **
PD	0.20 ***	−0.54 ***	−0.05	−0.06	−0.08	0	−0.09	−0.15 **	1

*Note.* TP, trust in the police; TPA, trust in the parliament; TE, trust in the educational system; TC, trust in the court system; TA, trust in the army; PD, psychological distress. * *p* < 0.05; ** *p* < 0.01; *** *p* < 0.001.

**Table 3:** The explanation of psychological distress among the two groups.

	Jews					Arabs				
	*R* ^2^	B	*Β*	SE	t	*R* ^2^	B	*Β*	*SE*	*t*
**Step 1**	0.16					0.01				
Gender		0.42	0.30	0.08	5.23 ***		0.04	0.02	0.16	0.28
Age		−0.01	−0.25	0.00	−4.36 ***		−0.01	0.08	0.01	−0.92
Socioeconomic status		−0.05	−0.05	0.05	−0.90		−0.02	−0.02	0.13	−0.19
**Step 2**	0.03					0.01				
Exposure		0.68	0.19	0.21	3.25 ***		−0.47	−0.08	0.50	−0.94
**Step 3**	0.04					0.09				
Trust: Army		−0.08	−0.14	0.04	−2.24 *		0.04	0.09	0.07	0.59
Trust: Parliament		−0.02	−0.04	0.04	−0.53		−0.14	−0.25	0.07	−1.96 ˆ
Trust: Media		0.08	0.17	0.04	2.26 *		−0.03	−0.04	0.08	−0.34
Trust: Police		−0.02	−0.04	0.04	0.51		0.08	0.16	0.07	1.10
Trust: Educational system		−0.02	−0.04	0.04	0.51		0.10	0.17	0.07	1.37
Trust: Court system		−0.02	−0.06	0.03	−0.76		0.06	0.12	0.07	0.91
**Step 4**	0.17					0.22				
SOC		−0.43	0.45	0	−8.38 ***		−0.60	−0.51	0.10	−6.13 ***
**Total**	0.40					0.33				

ˆ *p* = 0.05; * *p* < 0.05; *** *p* < 0.001.

## Data Availability

The data collected and used in this research are available from the authors upon reasonable request.

## Abbreviation

The following abbreviation is used in this manuscript:

SOC	Sense of coherence
